# Protocol for a non-surgical model of perineural invasion for assessing neural drivers of cancer aggressiveness in mice

**DOI:** 10.1016/j.xpro.2024.103345

**Published:** 2024-09-26

**Authors:** Tiago H. Zaninelli, Telma Saraiva-Santos, Brielle H. Patlin, Michael Slade Stratton, David Y. Chen, Felipe A. Pinho-Ribeiro

**Affiliations:** 1 Division of Dermatology, Department of Medicine, Washington University School of Medicine in St. Louis, Saint Louis, MO, USA

**Keywords:** Cancer, Model Organisms, Neuroscience

## Abstract

Perineural invasion (PNI) is a significant risk factor for cancer recurrence and metastasis; however, its mechanisms relating to cancer aggressiveness remain poorly understood. Here, we present a protocol for a non-surgical model of PNI in mice using a neurotropic melanoma cell line that migrates from the skin to the sciatic nerve. We describe the steps for cell culture and injection, tumor burden measurements, mouse euthanasia, and tissue dissection. We then detail procedures for sample cross-section and confocal imaging.

## Highlights

• B16F10-Fluc-Neo/eGFP-Puro culture and expansion
• Mouse injection site and tumor cell inoculum preparation
• Non-surgical perineural invasion model by subcutaneous injection
• Bioluminescence tumor growth monitoring and sample processing

## Abstract (editor highlights)

Publisher’s note: Undertaking any experimental protocol requires adherence to local institutional guidelines for laboratory safety and ethics.

## Graphical abstract

**Figure:**
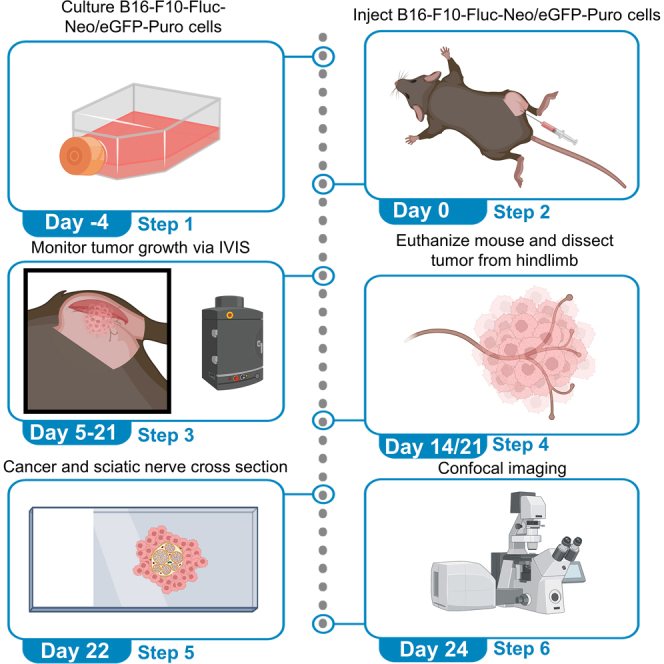

## Before you begin

### Overview

This protocol describes a non-surgical, pre-clinical model of skin cancer perineural invasion (PNI) in mice using a bioluminescent and fluorescent melanoma cell line. PNI occurs when cancer cells infiltrate and encase peripheral nerve fibers. In this process of metastasis, once invading the nerve shaft, tumor cells are less susceptible to the immune system and exploit nerve-derived factors that may increase tumor cell aggressiveness and survival.^1^ PNI is a clinically significant negative prognostic indicator, signaling an increased risk of local recurrence and metastatic disease.^1^^,^^2^^,^^3^ PNI also contributes significantly to patient suffering by causing severe chronic pain and sensory dysfunction.^4^^,^^5^^,^^6^ Despite being acknowledged as a hallmark of aggressive cancers, PNI remains underexplored and inadequately addressed by current therapies.^3^ The sciatic nerve model is commonly used to study how cancer cells thrive within nerves. However, this model is limited because it involves directly introducing cells into the nerve, bypassing the early stages of PNI.^7^ Previous studies *in vitro* suggest that molecular interactions between nerves and tumor cells occur before physical contact.^7^ Yet, the specific molecular mechanisms driving neurotropism and the aggressiveness of nerve-interacting cancer cells remain poorly understood. This gap in knowledge hinders the development of targeted therapies and adversely affects disease outcomes.

To bridge this gap, we have developed an *in vivo* PNI model that allows studying the interactions between cancer cells and nerves from the early stages before nerve invasion occurs. We propose a non-surgical pre-clinical model using B16F10-Fluc-Neo/eGFP-Puro cells, a well-established experimental model system arising from melanoma in B6 mice. This protocol involves injecting cancer cells subcutaneously into the popliteal fossa of the mouse. The cells then migrate through connective tissue and muscle layers to robustly interact with the sciatic nerve, closely mimicking the histologic features of PNI in human skin cancers. The development of PNI and the progression of cancer can be easily determined by *in vivo* bioluminescence imaging, flow cytometry, and microscopy.

This model not only underscores the pronounced neurotropism of a skin cancer cell line but also showcases its ability to rapidly initiate PNI in a murine model. Furthermore, this approach facilitates the observation of PNI’s effects on tumor progression and indicates that nerve-interacting cancer cells acquire an enhanced capacity for growth and invasion. Thus, this protocol serves as a powerful pre-clinical tool to uncover the mechanisms that drive the aggressive nature of PNI-developing cancers and to identify new therapeutic targets and strategies to combat cancer metastasis, recurrence, and sensory impairment.

### Institutional permissions

All experiments on mice were performed under the approval of the Institutional Animal Care and Use Committee (IACUC) from Washington University School of Medicine in Saint Louis under protocol number 22–0148. Researchers utilizing this protocol are required to acquire authorization and training from their institution’s Animal Ethics Committee.

## Key resources table

**Table:** 

REAGENT or RESOURCE	SOURCE	IDENTIFIER
**Biological samples**		

Tumor	This study	N/A
Sciatic nerve	This study	N/A

**Chemicals, peptides, and recombinant proteins**		

D-luciferin	Gold Biotechnology	Cat# 115144359
DMEM high glucose, w/ L-glutamine w, sodium pyruvate	Genesee Scientific	Cat# 25-500
Fetal bovine serum (FBS)	Sigma Chemical	Cat# F2442
Trypsin-EDTA solution	Sigma Chemical	Cat# T4049
Penicillin-Streptomycin	Gibco	Cat# 15140122
Trypan blue solution	Corning	Cat# 25900CL
Phosphate-buffered saline, pH 7.4	Sigma Chemical	Cat# D8537-6x1
Heparin	Fisher Scientific	Cat# ICN19411450
Paraformaldehyde solution, 4% in PBS	Thermo Fisher Scientific	Cat# 10010023
Sucrose	Sigma-Aldrich	Cat# 55016
O.C.T Compound	Fisher Health Care	Cat# 4585
Normal donkey serum	Abcam	Cat# ab7475
Fluoromount-G mounting medium, with DAPI	Invitrogen	Cat# 00-4959-52

**Experimental model: Cell line**		

B16F10-Fluc-Neo/eGFP-Puro	Imanis Life Sciences	Cat# CL068

**Experimental model: Organism**		

C57BL/6J mice (female and male), 6–8 weeks old	The Jackson Laboratory	Cat# 000664
Nav1.8-cre (B6.129(Cg)-Scn10atm2(cre)Jwo/TjpJ) (female and male), 6–8 weeks old, breeding propose	The Jackson Laboratory	Cat# 036564
Ai9 (B6.Cg-Gt(ROSA)26Sortm9(CAG-tdTomato)Hze/J) (female and male), 6–8 weeks old, breeding propose	The Jackson Laboratory	Cat# 007909
Nav1.8-tdTomato, (female and male), 6–8 weeks old	F1 litters from Nav1.8-cre and Ai9 breeding pars	N/A

**Software**		

Living Image 2.6	Xenogen	N/A
LAS X software	Leica Microsystems	N/A
Adobe Illustrator CC	Adobe	N/A

**Other**		

15- and 50-mL centrifuge tubes	MidSci	Cat# C15B and C50B
T-175 flasks for cell culture	Thermo Fisher Scientific	Cat# 159920
CO2 incubator	Fisher Scientific	Cat# 1078684
Neubauer chamber	Fisher Scientific	Cat# 15980396
Centrifuge Sorvall × 4R	Thermo Fisher Scientific	Cat# 75009900
Depilatory cream	Nair	N/A
Cotton-tipped applicators	McKesson	Cat# 241062S
Heating pad	Bilt-Rite Mastex Health	N/A
IVIS 50	PerkinElmer	N/A
10 mL syringe	Fisher Scientific	Cat#14955495
1 mL Insulin syringe	Exel International	Cat# 26027
Surgical scissors	N/A	N/A
Forceps	N/A	N/A
Tissue-Tek Cryomold molds	Sakura Finetek	Cat# 25608916
Cryostat CM1950	Leica	N/A
PAP pen	Newcomer Supply	Cat# 6506
Slide tray for immunostaining	Research Products International Corp	CAT# 195801
Triton X-100	Sigma-Aldrich	Cat# 9036-19-5
Microscopy slides charged (25 × 75 × 1 mm)	Globe	Cat# 1358P
Microscope cover glass (24 × 60 mm)	Globe	Cat# 141910
Stellaris 8 Sted microscope	Leica	N/A
Digital Vernier Caliper 6 inch/150 mm	Proster	N/A
Gram scale small digital scale	Skeap	N/A

## Materials and equipment

**Table:** Culture medium

Reagent	Final concentration	Amount
DMEM High Glucose, w/ L-Glutamine w, Sodium Pyruvate	89%	445 mL
FBS	10%	50 mL
Penicillin-Streptomycin	1%	5 mL
Total	–	500 mL

Store at 4°C for up to two weeks.

### Other solutions

• **Sucrose 30%:** Prepare 30% (w/v) sucrose in 1× PBS solution. Sterilize the solution by filtering it in a 0.22 μm membrane filter and storing it at 4°C. To maintain sterility, open the sucrose solution flask in a biosafety cabinet.
• **70% Ethanol:** Prepare 70% (v/v) ethanol in de-ionized water.
• **PBSt 0.3%:** Prepare 0.3% (v/v) Triton x-100 in 1× PBS solution.
• **D-Luciferin stock solution:** Dissolve 1 g D-Luciferin in 33.33 mL of PBS (30 mg/mL).

***Note:*** Store at −20°C as 1 mL aliquots for up to 6 months.

### Isoflurane anesthesia

• 3% isoflurane (set in the vaporizer).
• Oxygen flow rate of 1.5 L per minute (LPM).

### *In vivo* imaging system

• *In vivo* bioluminescence imaging equipment IVIS 50 In Vivo Bioluminescence Imaging System and Living Image 2.6 software (PerkinElmer, Waltham, MA, USA) with the following settings:
  ○ 10 s of exposure time, 8 bin, FOV13.3 cm, f/1, open filter.
  ○ Using the Living Image software, measure the total photon flux (photons/sec) from a user-defined circle (region of interest, ROI) with a 2 cm diameter over the right hindlimb/flank.

### Confocal microscopy

• Confocal microscope Stellaris 8 Sted (Leica, Wetzlar, Germany) with the following settings:
  ○ DAPI
    - Laser Will 80% power, Excitation 360 nm, 10% Smart Power.
    - Hybrid Detector 1, 400–470 nm spectral filter, Smart Gain 2.5–50%.
  ○ B16F10-eGFP
    - Laser Will 80% power, Excitation 488 nm, 10% Smart Power.
    - Hybrid Detector 2, 500–570 nm spectral filter, Smart Gain 2.5%–50%.

### Cryostat

Cryostat CM1950 (Leica, Wetzlar, Germany) with the following settings.

• Temperature: −20°C.
• Step size: 20 μm.

## Step-by-step method details

### Step 1: Cell culture

**Timing: 4 days**

This step consists of the melanoma cell line B16F10-Fluc-Neo/eGFP-Puro cell culture and expansion for the non-surgical induction of PNI in mice.

1. Thaw a vial of B16F10-Fluc-Neo/eGFP-Puro cells (∼2.8 × 10^6^) in tissue-culture treated surface culture flask T175 by adding 20 mL of DMEM containing L-glutamine, 10% FBS, and 1% Penicillin-Streptomycin.
2. Incubate the cells at 37°C and 5% CO2 (v/v) for 48 h or until they reach 85%–90% confluency.
3. Wash the flask thoroughly two times with sterile PBS.
4. Incubate cells with 5 mL of pre-warmed Trypsin solution for 5 min or until all cells detach from the flask.
5. Add 5 mL of complete DMEM to inactivate the trypsin.
6. Aspirate the cell suspension and transfer it into a conical 50 mL centrifuge tube.
7. Centrifuge cells (5 min, 300 × *g*, 4°C).
8. Resuspend the cell pellet in 40 mL of complete DMEM and split the volume into two T175 culture flasks.
9. Incubate the cells at 37°C and 5% CO2 (v/v) for 48 h or until they reach 85%–90% confluency.

### Step 2: Injection site preparation

**Timing: 20 min/mouse**

**Figure 1:**
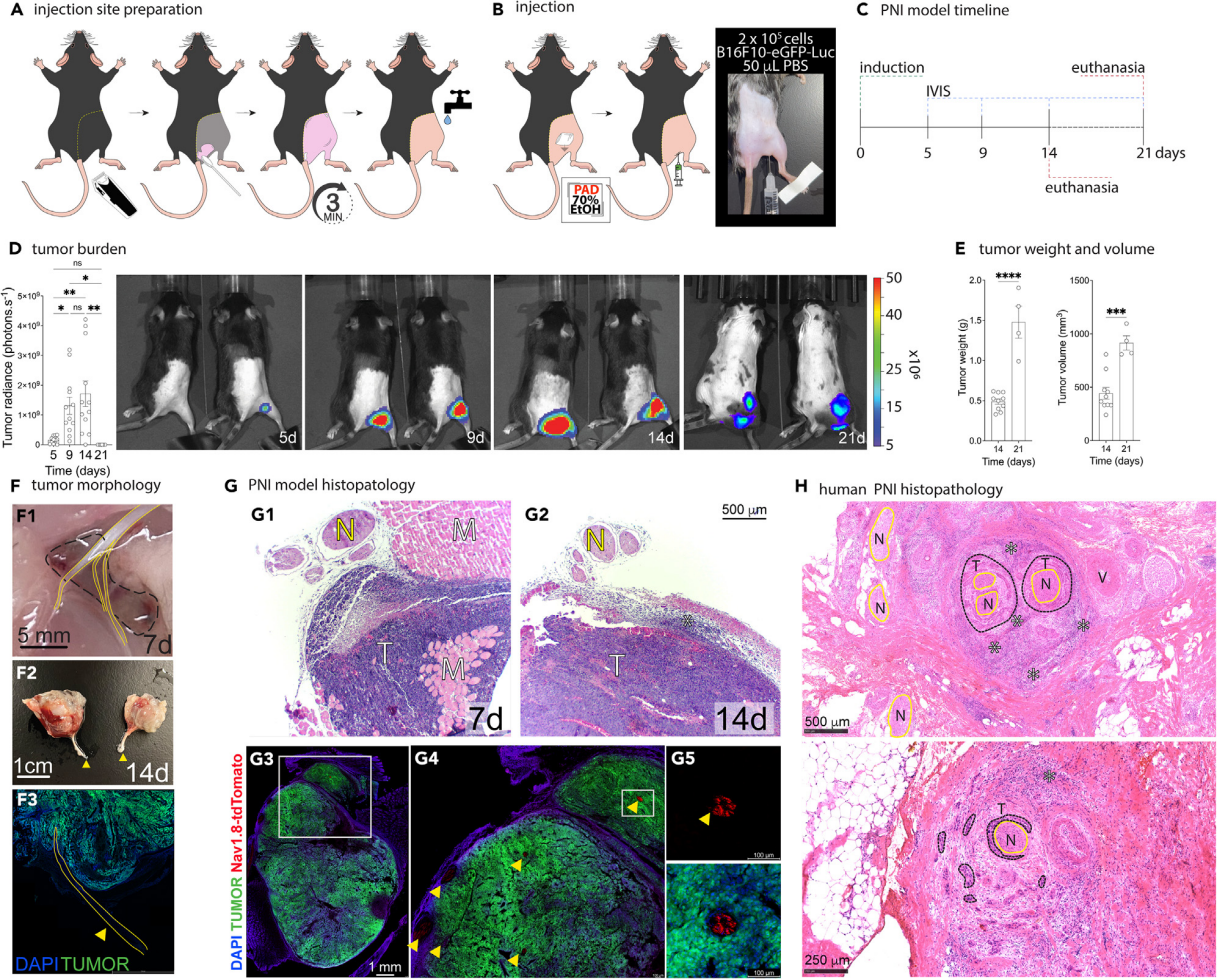
Non-surgical PNI model: injection site preparation, model induction, and outcomes (A) Injection site preparation. (B) PNI model induction by subcutaneous injection of B16F10-Fluc-Neo/eGFP-Puro melanoma cells. (C) Model experimental procedures and timeline. (D) Tumor burden assessment by *in vivo* bioluminescence imaging and representative images at 5, 9, and 14 days after tumor injection. ∗*p* < 0.05, ∗∗*p* < 0.005. Colorimetric scale indicates number of photons × 10^6^. (E) Tumor weight and volume at 14 days after injection. ∗∗∗∗*p* < 0.0001, ∗∗∗*p* = 0.0004. (F1) A representative image shows the establishment and growth of tumors deep into the muscle layers by migrating cancer cells 7 days after skin injection. The sciatic nerve is outlined in yellow, and the tumor is outlined with a dashed black line. Scale bar corresponds to 5 mm. (F2) Macroscopic view of dissected tumors and sciatic nerve. Yellow arrowhead points to the sciatic nerve. Scale bar corresponds to 1 cm. (F3) Longitudinal section of tumor and sciatic nerve highlighted in yellow. Scale bar corresponds to 1 cm. (G) Representative image of PNI model (G1-G2) histopathology (N – nerve, M – muscle, T – tumor, ∗ - inflammatory infiltrate, scale bar corresponds to 500 μm) and (G3-G5) immunohistochemistry cryosections. Green = tumor cells (eGFP+). Blue = cell nuclei (DAPI). Red = sciatic nerve sensory fibers (Nav1.8-tdTomato). G3 scale bar corresponds to 1 mm, G4-G5 scale bars correspond to 100 μm. Gray squares in G3 and G4 show the regions magnified to generate G4 and G5, respectively; yellow arrowheads point to nerve fibers. (H) Histopathology of skin cancer PNI in humans (N – nerve [yellow outline], T – tumor [dashed black outline], V – vein, ∗ - inflammatory infiltrate, scale bars are indicated as 500 μm [top] and 250 μm [bottom]).

This step accomplishes preparing the skin to allow proper visualization of the injection site and for bioluminescence imaging (Figure 1A).

10. Anesthetize mice using inhaling isoflurane 3% in O_2_ (v/v).
11. Place sterile ophthalmic ointment in both eyes to avoid corneal desiccation.
12. With the aid of a pet hair trimmer, carefully remove all hair around the right hindlimb and the medial-right dorsal region (Figure 1A).
13. Apply the depilatory cream using a cotton-tipped applicator to the trimmed region and let it act for 2–5 min (Figure 1A).
14. Wash the region thoroughly in warm running water, avoiding the face and ears (Figure 1A).

**CRITICAL:** Any remaining depilatory cream must be removed entirely. Prolonged contact with mouse skin may cause skin irritation.

15. Immediately after washing off the product, dry the animals with absorbent paper towels and place the mouse on a heated surface (∼37°C) for recovery (e.g., heating pad).

***Note:*** Only part of the recovery cage must be placed on the heating surface so the animals can choose the most comfortable temperature while returning from anesthesia.

16. Ensure the animals are dry and fully active before returning to their cages to the animal facility or moving to the next step.

### Step 3: B16-F10-Fluc-Neo/eGFP-Puro cell inoculum preparation

**Timing: 30 min**

Prepare the cell inoculum for the model induction.

17. Detach the B16-F10-Fluc-Neo/eGFP-Puro cells with the Trypsin solution.
  a. Remove culture media from both T175 flasks with a disposable 25 mL serological pipet.
  b. Using another disposable 25 mL serological pipet, add 10 mL of 1× sterile PBS in each flask.
  c. Carefully rinse all the flask’s culture surface by moving the flask in orbital movements.
  d. Remove the 1× PBS.
  e. Repeat steps a-d.
  f. Incubate cells with 5 mL of pre-warmed Trypsin-EDTA solution for 5 min or until all cells detach from the flask.
  g. Add 5 mL of complete DMEM to inactivate the trypsin in each flask.
  h. Aspirate the cell suspension from both flasks and pool them into a conical 50 mL centrifuge tube.
  i. Centrifuge cells (5 min, 300 × *g*, 4°C).
18. Remove the supernatant and resuspend the cell pellet in 5 mL of 1× sterile PBS.
19. Place the conical 50 mL centrifuge tube on ice.
20. In a 1.7 mL microtube, add 180 μL of 1× sterile PBS and 20 μL of the cell suspension obtained in the previous step.
21. In another 1.7 mL microtube, add 20 μL of Trypan Blue Solution 0.4% (w/v) and 20 μL of the cell suspension obtained in step 4.
22. Using a Hemocytometer (Improved Neubauer counting chamber), determine the number of cells per mL of solution.
  a. Add 10 μL of the solution obtained in step 5 into the two counting fields of the chamber.
  b. Count the number of live cells (i.e., negative for Trypan Blue staining) in the four primary quadrants (corners) in both counting fields.
  c. Use the following formula to determine the cell number per mL.
    $$X=\frac{\left(c1+c2\right)}{2}$$
    $$Y=\frac{X}{4}\times 20\times 10\times 1000$$
    X – average of cells in the two fields from the counting chamber
    c1 – number of cells in the field 1 of the chamber
    c2 - number of cells in the field 2 of the chamber.
    Y – number of cells per mL of solution.
23. Adjust the number of cells to a final concentration of 2 × 10^5^ cells per 50 μL.
  a. Use the following formula to prepare the inoculum:
    $$Z=\frac{\left(200\times 10^{6}\right)}{Y}$$
    Z – volume needed from cell suspension (Y) for an inoculum of 2 × 10^5^ cells per mouse.
    Y – number of cells per mL of solution from step 6c.
  b. Adjust the volume of the final inoculum to 50 μL by adding sterile 1× PBS.
    ***Note:*** In case the value of Z is superior to 50 μL, concentrate the cells by centrifuging (5 min, 300 × *g*, 4°C), resuspending the pellet in 1 mL of sterile 1× PBS, and repeating steps 4–8.
24. After cell concentration and volume adjustment, keep the inoculum on ice until the mice are injected.

**CRITICAL:** The time between inoculum preparation and animal injection should not exceed 1 hr.

### Step 4: Subcutaneous injection

**Timing: 4 min/mouse**

Induction of the non-surgical model of melanoma PNI.

25. Anesthetize the mouse with isoflurane (3% v/v in O_2_) and maintain the anesthesia during the entire process.
26. Using a micropipette, transfer 50 μL of the cell inoculum into a 1.7 mL microcentrifuge tube cap.
27. Load the insulin syringe by aspirating the volume in the microtube’s cap; remove air bubbles.
28. Place the animal in the ventral position and fix the animal’s rear limbs using adhesive tape.
29. Disinfect the injection site with 70% ethanol pads.
30. Position the syringe parallel to the bench surface and the needle perpendicular to the skin into the popliteal fossa (Figure 1B).

***Note:*** Insert 2 mm of the needle with the bevel facing up and inject the inoculum (Figure 1B).

31. Wait 5 s after injection, and then carefully remove the needle.

**Figure 2:**
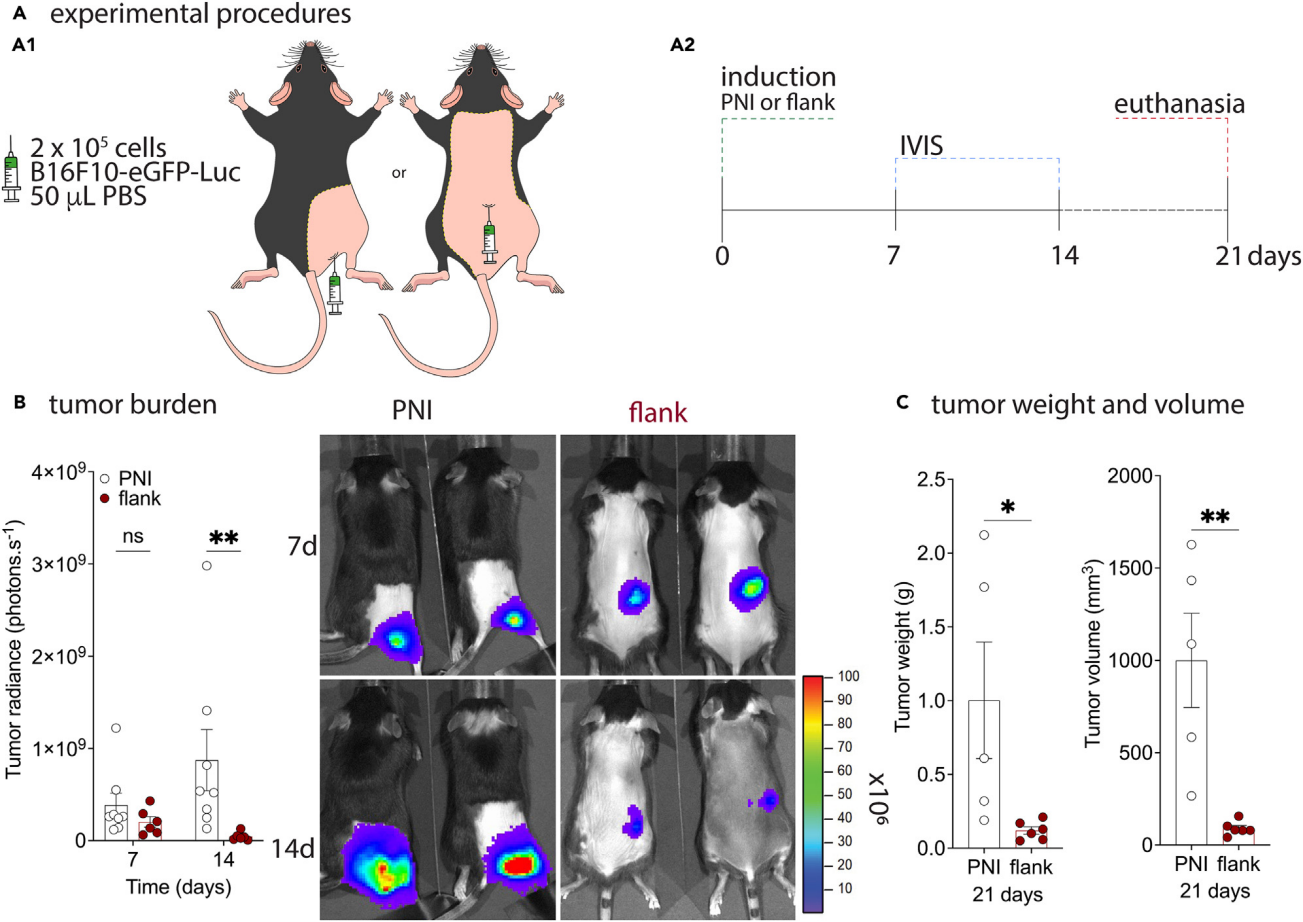
Proximity to the sciatic nerve sustains and increases tumor growth (A) Mice were injected subcutaneously with 2 × 10^5^ B16F10-Fluc-Neo/eGFP-Puro melanoma cells in the right rear limb (PNI) or right flank. (B) After injection, tumor burden was determined by *in vivo* imaging and at the time points of 7 and 14 days. Colorimetric scale indicates number of photons × 10^6^. ∗∗*p* = 0.0099. (C) On the 21^st^ day after injection, the tumor was dissected, weighted, and measured for volume determination. ∗*p* = 0.0358, ∗∗*p* = 0.0034.

***Note:*** the site of injection is critical to PNI model success. Tumor growth kinetics is increased in proximity to the sciatic nerve in comparison to other injection sites (Figure 2).

32. Remove the mouse from anesthesia and place it on a cage containing a heating pad (37°C) for recovery.

***Note:*** Only part of the recovery cage must be placed on the heating surface so the animals can choose the most comfortable temperature when returning from anesthesia.

33. Ensure the animals are fully active before returning to their cages.

### Step 5: Tumor burden monitoring

**Timing: 11 min/mouse**

Tumor growth evaluation by *in vivo* bioluminescence imaging using IVIS 50 and Living Image 4.3.1 (PerkinElmer, Waltham, MA). Measurements begin on day 5 post-injection. For tumor burden measurements we recommend the following time points: 5, 9, and 14 days, or 7 and 14 days post-injection.

34. Ten minutes before imaging, intraperitoneally administer 10 μL of D-luciferin (150 μg/mL in PBS; Gold Biotechnology, St. Louis, MO) per gram of animal weight (e.g., for a mouse weighting 20 g, inject 200 μL of deliver D-luciferin [150 μg/mL], corresponding to a dose of 150 mg/kg).
35. Anesthetize the mouse with isoflurane (3% v/v in O_2_) and place it inside the equipment.
36. Within the completion of 10 min, image the mice using the following configuration: 10 s of exposure time, 8 bin, FOV12cm, f/stop1, open filter (Figure 1D).
37. After the acquisition, return the animal to its cage.
38. Quantify the radiance of each mouse using the software Living Image 4.3.1 (Figure 1D).
39. Tissue radiance values are presented as photons per second (photons.sec^−1^).

***Note:*** Bioluminescence values and tumor size change linearly up to day 14 post-injection time point (Figures 1D and 1E). As the tumor continues to grow, the measurement of luciferase reaction is negatively impacted on day 21 due to the development of necrosis and impaired blood perfusion in the tumor.

### Step 6: Dissection and processing of samples of tumor PNI post-mortem (terminal)

**Timing: 20 min/mouse**

40. Prepare the exsanguination station with the following materials:
  a. 10 mL syringe.
  b. 30-gauge (G) needle.
  c. Surgical scissors.
  d. Forceps.
41. Prepare a 1× PBS solution with 0.2% heparin and store it at 4°C.

***Note:*** The required volume per mouse is 10 mL.

42. Load a 10 mL syringe with 1× phosphate-buffered saline (PBS) with 0.2% of heparin, attach the 30G needle, remove all air bubbles, and set aside at 4°C.
43. Load a 10 mL syringe with 4% Paraformaldehyde Solution (PFA), attach the 30G needle, remove all air bubbles, and set aside at 4°C.

***Note:*** The required volume per mouse is 10 mL.

44. Proceed with euthanasia using CO_2_ inhalation. It is important to perform this step at a pace of one mouse at a time to avoid blood clotting and impaired perfusion and fixation.
45. Initial incision:
  a. Ensure euthanasia by checking the animal’s vitals and responsiveness to toe pinch or other reflex tests before proceeding.
  b. Place the euthanized mouse on a dissection board in the supine position.
  c. Disinfect the thoracic site with 70% ethanol pads.
  d. Make a lateral incision below the sternum and rib cage to expose the diaphragm and liver.
46. Exsanguination and post-mortem fixative injection:
  a. Cut back the diaphragm using scissors and expose the pleural cavity and heart.
  b. Create a small incision in the right atrium.
  c. Identify the left ventricle and insert a 30-gauge needle with 1× PBS into the needle bevel.
  d. Once the blood has cleared from the circulatory system and the liver has lightened, switch the syringe to inject 4% PFA, until all tissues are fixed and appear pale.

***Note:*** For optimal results, maintain a slow and steady injection rate (around 5–10 mL/min) to avoid damaging the delicate tissues.

47. Sample collection:
  a. Once fixative injection is completed, use dissection scissors and forceps to make a midline incision through the skin on the mouse’s hind limb to expose the underlying muscles and nerves.
  b. Carefully separate the muscles and surrounding tissues to expose the tumor and sciatic nerve.
    ***Note:*** The sciatic nerve will be visible as a long, whitish, and cord-like structure (Figure 1F).
  c. Gently dissect and isolate the tumor and sciatic nerve from the surrounding tissues, careful not to damage or stretch the nerve.
    ***Note:*** Use micro-scissors (ophthalmic scissors) or other sharp surgical instrument for precise dissection.
  d. Once the tumor and sciatic nerve are exposed and isolated, carefully cut the nerve at the longitudinal and proximal areas.
  e. Determine the tumor weight using a calibrated precision scale (Figure 1E), and the volume by measuring the tumor length and width with a caliper ruler (Figure 1E).
  f. To determine tumor volume, use the following formula:
    $$\text{Tumor}\;\text{volume}=\text{length}\times {\left(\text{width}\right)}^{2}\times 0.5$$
48. PNI samples are then submerged in 4% PFA solution and incubated at 4°C for 24 h.

**CRITICAL:** Paraformaldehyde is a toxic substance that can lead to eye, respiratory, and dermatologic injuries. Procedures involving PFA should be performed by trained personnel following the institutional guidelines. PFA handling and disposal should be done very carefully, preferably inside appropriate chemical hoods, wearing personal protective equipment (PPE), and taking other safety measures as recommended by the institutional safety guidelines.

### Step 7: Processing and cryosection of tumor samples

**Timing: 3 days for steps 49 and 50, 50 min for step 51**

Process for cryoprotection and cryosection of mouse tumor tissue and sciatic nerve.

49. Wash the samples three times for 5 min each using 1× PBS.
50. Incubate samples with 30% sucrose solution at 4°C for 2 days.
  **CRITICAL:** The volume of solutions (1× PBS and 30% sucrose) should be at least 10× the tissue volume to achieve better results. Tissue initially floats in 30% sucrose solution but will eventually sink as the sucrose solution penetrates the tissue during the incubation.
  a. Transfer the samples to cryomolds filled with OCT. If needed, add more OCT so the tissue is completely immersed and not in contact with air. Put the tissue in the cryomolds with the sciatic nerve facing the bottom and freeze it in a 100 mL beaker immersed in liquid nitrogen containing 5–8 mL of isopentane in the bottom.
    **CRITICAL:** Careful handling and precise anatomic positioning of tumor samples in the cryomold during freezing are crucial for optimal cryosection.
    **Pause point:** Cryomolds containing the frozen tissues in OCT can be stored at −80°C or processed immediately as described in the following steps.
51. Cut transverse sections with 40 μm of thickness in the cryostat at −20°C and place the sections in a positively charged slide.

**Pause point:** The slides should be used after sectioning or stored at −20°C.

**CRITICAL:** To ensure proper tissue attachment to slides, it is important to keep slides at room temperature for a minimum of 30 min to allow tissue sections to melt and properly stick onto the slide before proceeding with assays or storing them in the freezer.

### Step 8: Immunofluorescence assay

**Timing: 2 days**

52. Slides are put in a humid slide chamber at room temperature, and gently draw a circle around the tissue sections with a PAP pen.
53. Rinse samples two times with 1× PBS for 10 min each.

***Note:*** Use the necessary volume of solution to cover the sections.

**CRITICAL:** If antibody staining is not required, skip to **step 9**.

54. If antibody staining is desired, block samples with a blocking solution or 5% bovine serum albumin in 1× PBS for 1 h at room temperature and proceed with the staining protocol most suitable for your antibody’s required conditions.
55. Mount slides using Fluoromount-G Mounting Medium with DAPI, cover with cover glass slides and store at 4°C protected from light.

***Note:*** Use the necessary volume of mounting medium to cover the sections.

**CRITICAL:** When applying the mounting media and coverslip, it is crucial to avoid trapping air bubbles, as they can interfere with imaging and cause artifacts.

**CRITICAL:** To maintain the slide's integrity and prevent drying, it is critical to seal the coverslip edges with nail polish or specialized sealing agents.

56. Proceed for sections imaging (Figures 1G3–1G5).

### Step 9: Confocal imaging

**Timing: 2 days**

57. Remove the slides from the 4°C and keep them at room temperature during the analysis.

**CRITICAL:** It is crucial to clean the slides before imaging to remove any contaminants, debris, or residues that could interfere with the imaging process. Even small particles or smudges can lead to unwanted background noise and reduce the clarity of your immunofluorescence signals. Gently wipe the slide's surface using a lint-free, non-abrasive cleaning cloth or lens paper. Wipe in a single direction to minimize the risk of introducing new particles or streaks.

58. Confocal microscope settings for Stellaris 8 Sted (Leica, Wetzlar, Germany).
  **CRITICAL:** Confocal microscopes are often assembled under request; therefore, laser and detectors will vary according to the maker and model. It is very important to consult the settings and be properly trained before image acquisition.
  a. DAPI.
    i. Laser Will 80%, Excitation 360 nm, 10% Smart Power.
    ii. Hybrid Detector 2, 400–470 nm spectral filter, Smart Gain 2.5–50%.
  b. B16F10-eGFP.
    i. Laser Will 80% power, Excitation 488 nm, 10% Smart Power.
    ii. Hybrid Detector 1, 500–570 nm spectral filter, Smart Gain 2.5%–50%.
  c. Nerve fibers – Nav1.8-tdTomato transgenic mice.
    i. Laser Will 80% power, Excitation 554 nm, 10% Smart Power.
    ii. Hybrid Detector 1, 580–690 nm spectral filter, Smart Gain 2.5–50%.
59. Objective lens 10×/0.4 dry (low magnification) or 20×/0.75 dry (higher magnification);
60. Use the tile tool to determine the perimeter of the tumor sciatic nerve section.
61. Perform Z-stack acquisition by capturing a series of optical sections along the Z-axis (2 μm per step size) to obtain a 3D sample representation.

## Expected outcomes

Tumor size and bioluminescence radiance present a linear trend up to 21 days after tumor injection. Involvement and proximity to sciatic nerve was observed in >90% of injected animals (*n* > 50). Tumor growth kinetics is increased in this model of PNI in comparison to the flank injection site (Figure 2). Those findings indicate that the proximity to the sciatic nerve sustains and increases tumor growth (Figures 2B and 2C).

## Limitations

At time points past 21 days after injection, the necrotic tissue drastically reduces the linearity of bioluminescence radiance associated with tumor growth and leads to mobility issues in the animal. The strong neurotropism of B16F10 cell line is a critical feature for the model of non-surgical PNI described here and may not be observed in other cell lines.

## Troubleshooting

### Problem 1

Wrong injection site or intradermal delivery of the cells (Step 4).

### Potential solution

Make sure the needle is placed in the subcutaneous space. Carefully angle the needle to the right and to the left to confirm it is not placed in the dermis.

### Problem 2

Leakage after the inoculation is strong evidence of non-successful injection (Step 4).

### Potential solution

• Using the index finger and thumb, pinch the injection site and feel the needle to confirm it is in the correct site, i.e., the subcutaneous space.
• Make sure about 2/3 of the needle is inside the tissue; avoid muscle tissue.

## Resource availability

### Lead contact

Felipe A. Pinho-Ribeiro (dfelipe@wustl.edu).

### Technical contact

Tiago H. Zaninelli (tiago@wustl.edu).

### Materials availability

This study did not generate new unique reagents.

### Data and code availability

This study did not generate/analyze datasets or codes.

## Acknowledgments

The authors thank Julie Prior and Katie Duncan for technical support with *in vivo* bioluminescence imaging. This work was supported in part by the following funding sources: 10.13039/100000002National Institutes of Health grants S10OD027042 and S10OD025264 and National Cancer Institute grant P30CA091842 to F.A.P.-R.; the 10.13039/100011912Washington University School of Medicine Faculty Diversity Scholars Award, Mallinckrodt Institute of Radiology Pilot Funding, and Institute of Clinical and Translational Sciences Just-In-Time Funding to F.A.P.-R.; the Elsa U. Pardee Foundation Research Grant to F.A.P.-R.; and the Chan Zuckerberg Initiative DAF and Silicon Valley Community Foundation (2024-338502) to F.A.P.-R.

## Author contributions

Conceptualization, F.A.P.-R. and T.H.Z.; supervision, F.A.P.-R. and D.Y.C.; resources, F.A.P.-R., D.Y.C., and M.S.S.; funding, F.A.P.-R.; methodology, T.H.Z. and T.S.-S.; writing – original draft, T.H.Z. and T.S.-S.; writing – review and editing, all authors. All authors have read and approved the final version of the manuscript.

## Declaration of interests

The authors declare no competing interests.
